# Delayed recognition of hypomagnesemia causing refractory Hypocalcemia after Reoperative neck surgery in a resource-limited setting. a case report

**DOI:** 10.1093/omcr/omag132

**Published:** 2026-07-27

**Authors:** Carlos Solórzano Flores, Sindy Michelle Vásquez, Andrea Pineda, Madison Rekken, Alejandra Laínez

**Affiliations:** Faculty of Medical Sciences, National Autonomous University of Honduras (UNAH), Calle La Salud, Edificio Administrativo, Tegucigalpa 11101, Honduras; Faculty of Medical Sciences, National Autonomous University of Honduras (UNAH), Calle La Salud, Edificio Administrativo, Tegucigalpa 11101, Honduras; Faculty of Medical Sciences, Central American Technological University (UNITEC), Kennedy Boulevard, V-782, Tegucigalpa 11101, Honduras; Faculty of Medical Sciences, Central American Technological University (UNITEC), Kennedy Boulevard, V-782, Tegucigalpa 11101, Honduras; Faculty of Medical Sciences, National Autonomous University of Honduras (UNAH), Calle La Salud, Edificio Administrativo, Tegucigalpa 11101, Honduras

**Keywords:** endocrinology and metabolism

## Abstract

Postoperative hypocalcemia following cervical surgery is typically attributed to hypoparathyroidism. However, hypomagnesemia is a reversible cause that impairs parathyroid hormone (PTH) secretion and target-organ responsiveness. We report a 35-year-old woman with recurrent symptomatic hypocalcemia after reoperative neck exploration for recurrent papillary thyroid carcinoma. Despite aggressive calcium and vitamin D supplementation, hypocalcemia persisted with inappropriately suppressed PTH. Due to resource limitations, serum magnesium evaluation was delayed until postoperative day 6, revealing severe hypomagnesemia. Magnesium repletion led to prompt serum calcium improvement and partial PTH recovery. Although the lack of contemporaneous phosphate and serial PTH measurements is a limitation, the rapid clinical response strongly supports a functional hypoparathyroid state. This case emphasizes early magnesium assessment in refractory postoperative hypocalcemia, demonstrating how resource limitations can delay diagnosis and prolong morbidity.

## Introduction

Postoperative hypocalcemia is the most common complication following thyroid surgery and is usually attributed to transient hypoparathyroidism caused by surgical manipulation, devascularization, or inadvertent injury to the parathyroid glands [1]. While most cases are self-limited and respond to calcium and vitamin D supplementation, persistent or refractory hypocalcemia should prompt evaluation for additional contributing mechanisms.

Magnesium plays a critical role in calcium homeostasis and is required for both parathyroid hormone (PTH) secretion and peripheral responsiveness [2]. Severe hypomagnesemia can suppress PTH release and induce target-organ resistance, producing a biochemical profile that mimics hypoparathyroidism [3]. Despite this, it remains an underrecognized cause of refractory postoperative hypocalcemia.

We report a case of persistent hypocalcemia following reoperative cervical surgery in which delayed recognition of severe hypomagnesemia was the primary reversible factor, highlighting the importance of early magnesium assessment, particularly in resource-limited settings.

## Case presentation

A 35-year-old woman with prior total thyroidectomy for papillary thyroid carcinoma (2023), not treated with radioactive iodine, was evaluated for suspected locoregional recurrence. She was maintained on levothyroxine 150 μg daily and had no comorbidities.

Neck ultrasonography demonstrated a 9.9 × 4.0 mm thyroid bed lesion and multiple abnormal right cervical lymph nodes (levels IV–VB–VA), suspicious for metastatic disease. Preoperative serum calcium was 8.8 mg/dL, at the lower limit of the normal range. TSH was mildly elevated (4.25 mIU/l) and thyroglobulin was 16.9 ng/ml.

The patient underwent reoperative cervical lymph node dissection without intraoperative complications. Histopathology confirmed metastatic papillary thyroid carcinoma. Parathyroid glands were preserved, although devascularization could not be excluded.

On postoperative day (POD) 1, she developed symptomatic hypocalcemia (paresthesias, carpopedal spasm, positive Chvostek and Trousseau signs) with serum calcium of 6.62 mg/dL. She was treated with intravenous calcium gluconate (2 g bolus followed by continuous infusion of 8 g/24 h, equivalent to ~ 720 mg elemental calcium/day), together with high-dose of oral calcium citrate (up to ~ 3.6 g elemental calcium/day) and vitamin D supplementation.

Despite escalating therapy over the first postoperative days, hypocalcemia remained refractory, with persistent serum calcium values between 6.48–7.36 mg/dl.

On POD 5, parathyroid hormone was inappropriately suppressed at 3.71 pg/ml in the setting of hypocalcemia, while 25-hydroxyvitamin D was normal (30.06 ng/ml), initially suggesting postoperative hypoparathyroidism. Oral cholecalciferol (25 000 IU) was administered in addition to ongoing calcium therapy.

Serum magnesium measurement became available on POD 6 due to laboratory limitations and was markedly reduced at 1.0 mg/dl, consistent with severe hypomagnesemia contributing to functional hypoparathyroidism.

Magnesium replacement was initiated with oral magnesium chloride (~1.4 g elemental magnesium/day), later escalated to magnesium lactate and increased total supplementation up to ~ 2.3 g elemental magnesium/day. Calcium supplementation was maintained with oral calcium carbonate (~720 mg elemental calcium/day) and ongoing intravenous calcium support as needed.

Following magnesium repletion, progressive biochemical improvement was observed, with partial recovery of PTH by POD 9 and normalization of magnesium by POD 11 (1.8 mg/dl). This was accompanied by normalization of serum calcium (8.27 mg/dl) and complete resolution of symptoms.

The patient was discharged on oral calcium, alfacalcidol 0.25 mg twice daily, cholecalciferol 100 000 UI weekly for four weeks, and maintenance oral magnesium supplementation.

**Figure 1:**
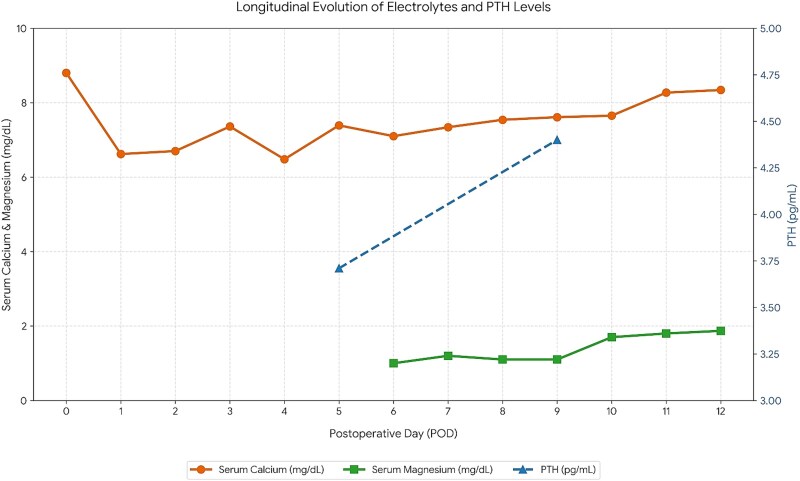
Longitudinal evolution of serum calcium, magnesium and parathyroid hormone (PTH) levels during the postoperative period. Persistent hypocalcemia and suppressed parathyroid hormone (PTH; nadir 3.71 pg/mL) remained refractory to calcium and vitamin D supplementation during postoperative days (PODs) 1–6. Magnesium replacement was initiated on POD 6, after which serum magnesium and calcium levels increased concurrently, accompanied by a rise in PTH levels (4.40 pg/mL). The temporal association between correction of hypomagnesemia and normalization of calcium levels supports the role of magnesium depletion in the development of functional hypoparathyroidism and impaired PTH responsiveness in this patient. Serum phosphorus measurements were unavailable because of a temporary shortage of laboratory reagents.

Temporal trends in serum calcium and magnesium concentrations during hospitalization are shown in Fig. 1.

## Discussion

Postoperative hypocalcemia is the most common complication following thyroid surgery, occurring transiently in up to 30%–40% of patients, whereas permanent hypoparathyroidism develops in approximately 1%–3% of cases. The primary mechanism is hypoparathyroidism resulting from surgical manipulation, devascularization, or inadvertent removal of the parathyroid glands, with risk increasing in reoperative procedures and lymph node dissection [4–6]. Furthermore, the identification of a base-line low normal serum calcium level during the preoperative evaluation serves as a crucial early predictor for subsequent hypocalcemia, a vulnerability that was clearly in this patient.

In the present case, the patient developed severe and persistent hypocalcemia immediately following reoperative cervical surgery despite aggressive calcium and vitamin D supplementation. The presence of inappropriately suppressed PTH levels (3.71 pg/ml) in the setting of significant hypocalcemia initially suggested postsurgical hypoparathyroidism. However, the clinical course was atypical, as hypocalcemia remained refractory despite appropriate therapy.

The subsequent identification of severe hypomagnesemia and the rapid biochemical response following magnesium replacement strongly support a reversible functional hypoparathyroid state. Magnesium plays a central role in calcium homeostasis by regulating both PTH secretion and end-organ responsiveness. Severe hypomagnesemia impairs cyclic adenosine monophosphate–mediated PTH release and induces peripheral resistance to PTH, resulting in hypocalcemia that is often refractory to calcium and vitamin D therapy alone [7, 8]. Although the patient’s serum 25-hydroxyvitamin D was normal, alfacalcidol was administered because magnesium deficiency can impair 1-alpha hydroxylase activity, limiting conversion of vitamin D to its biologically active form. This mechanism likely contributed to the persistence of hypocalcemia until magnesium repletion [9].

In this patient, the delayed measurement of serum magnesium limited early recognition of this mechanism. Although the precise onset of hypomagnesemia cannot be determined, it likely contributed to both the severity and persistence of hypocalcemia. The rapid normalization of calcium levels following magnesium repletion, together with partial recovery of PTH, supports a causal relationship.

The etiology of the severe hypomagnesemia is likely multifactorial. Contributing factors may include reduced oral intake in the postoperative period, intracellular shifts related to surgical stress, and renal magnesium losses associated with perioperative fluid administration and electrolyte disturbances [10]. While no single mechanism can be definitively established, these factors likely acted in combination. The comprehensive clinical spectrum and risk factors of acquired hypomagnesemia are detailed in Table 1 [10–13].

**Table 1:** Clinical Etiologies and pathophysiological mechanisms of acquired hypomagnesemia.

Site of Magnesium Loss or Dysregulation	Pathophysiological Mechanism	Clinical Etiologies and Triggers
Gastrointestinal tract	Impaired intestinal magnesium absorption due to altered TRPM6/TRPM7-mediated transport; binding of magnesium by unabsorbed fatty acids; direct loss of magnesium-rich gastrointestinal secretions [10–13].	Long-term proton pump inhibitor therapy; celiac disease; Crohn’s disease; short bowel syndrome; chronic alcohol use disorder; severe acute pancreatitis [10–13].
Renal tubules (drug-induced losses)	Disruption of the lumen-positive electrical gradient in the thick ascending limb of Henle and direct tubular toxicity impairing magnesium reabsorption; downregulation of epidermal growth factor signaling [11–13].	Loop diuretics; thiazide diuretics; cisplatin; cetuximab; aminoglycosides; amphotericin B; cyclosporine; tacrolimus [11–13].
Renal tubules (metabolic/intrinsic causes)	Increased tubular flow and impaired magnesium reabsorption associated with osmotic diuresis or transient tubular dysfunction [10, 11].	Uncontrolled diabetes mellitus with glycosuria; recovery phase of acute tubular necrosis; post-obstructive diuresis [10, 11].
Bone and systemic shifts	Redistribution of magnesium into newly mineralizing bone or intracellular compartments [10].	Hungry bone syndrome; refeeding syndrome [10].

A key limitation of this case is the absence of serial PTH and serum phosphate measurements during the phase of magnesium repletion. These parameters could have provided additional biochemical confirmation of functional hypoparathyroidism and facilitated differentiation from true postoperative hypoparathyroidism. Nevertheless, the temporal association between magnesium correction and normalization of calcium levels provides strong indirect evidence supporting this mechanism.

This case underscores several clinically important considerations. Persistent or refractory hypocalcemia following thyroid surgery should prompt early evaluation of serum magnesium, particularly when PTH levels are inappropriately low or fail to respond to standard therapy. Failure to recognize hypomagnesemia may lead to prolonged hospitalization, unnecessary escalation of calcium and vitamin D therapy, and misclassification of patients as having permanent hypoparathyroidism.

An additional important aspect is the impact of healthcare resource limitations. In this case, delayed access to serum magnesium measurement contributed to prolonged symptomatic hypocalcemia and extended hospitalization. In resource-limited settings, lack of timely diagnostic testing may significantly influence clinical outcomes and delay appropriate management.

In conclusion, severe hypomagnesemia should be considered a reversible cause of impaired PTH secretion and action in patients with persistent hypocalcemia after thyroid surgery. Early assessment of serum magnesium is essential to ensure timely diagnosis, guide appropriate therapy, and prevent avoidable morbidity.
